# Increased spontaneous MEG signal diversity for psychoactive doses of ketamine, LSD and psilocybin

**DOI:** 10.1038/srep46421

**Published:** 2017-04-19

**Authors:** Michael M. Schartner, Robin L. Carhart-Harris, Adam B. Barrett, Anil K. Seth, Suresh D. Muthukumaraswamy

**Affiliations:** 1 Sackler Centre for Consciousness Science, University of Sussex, Informatics Department, Brighton, BN1 9QJ, UK; 2 Psychedelic Research Group, Centre for Psychiatry, Division of Brain Science, Imperial College London, W12 0NN, UK; 3 School of Pharmacy, School of Psychology, University of Auckland, Auckland, 1010, New Zealand

## Abstract

What is the level of consciousness of the psychedelic state? Empirically, measures of neural signal diversity such as entropy and Lempel-Ziv (LZ) complexity score higher for wakeful rest than for states with lower conscious level like propofol-induced anesthesia. Here we compute these measures for spontaneous magnetoencephalographic (MEG) signals from humans during altered states of consciousness induced by three psychedelic substances: psilocybin, ketamine and LSD. For all three, we find reliably higher spontaneous signal diversity, even when controlling for spectral changes. This increase is most pronounced for the single-channel LZ complexity measure, and hence for temporal, as opposed to spatial, signal diversity. We also uncover selective correlations between changes in signal diversity and phenomenological reports of the intensity of psychedelic experience. This is the first time that these measures have been applied to the psychedelic state and, crucially, that they have yielded values exceeding those of normal waking consciousness. These findings suggest that the sustained occurrence of psychedelic phenomenology constitutes an elevated level of consciousness - as measured by neural signal diversity.

Understanding the brain basis of consciousness remains one of the outstanding challenges in modern science. While rigorous definitions are still mainly lacking, consciousness can be defined rather broadly as that which “vanishes every night when we fall into dreamless sleep” and returns the next morning when we wake up^1^. Equally, when we are conscious, our conscious experiences are populated by a variety of perceptions, thoughts, and feelings that collectively form an integrated conscious scene. These observations lead to an intuitive distinction between conscious level (how conscious one is) and conscious content (what one is conscious of, when one is conscious). The large majority of recent neuroscientific research into consciousness has treated these dimensions separately^2^,^3^,^4^,^5^. Investigations of conscious level typically contrast global changes in brain activity among different states including wakeful awareness, various sleep stages, and different forms of anaesthesia. Many of these studies attempt to isolate neural changes that accompany alterations of conscious level independently of changes in general physiological arousal. Studies of conscious content have focused primarily on uncovering differences in brain activity between closely matched conscious and unconscious perception, while conscious level is maintained constant^6^.

Recently, following early suggestions that increased conscious level may be related to an increased range of conscious contents^3^,^7^, there has been growing interest in characterising how conscious level and conscious content may relate^2^,^5^. One empirical approach to this question is to apply emerging measures of conscious level to experimental manipulations that primarily affect conscious content. Here, we capitalise on the profound effects on conscious phenomenology elicited by psychedelic compounds, specifically LSD, psilocybin, and subanesthetic doses of ketamine. These drugs normally have profound and widespread effects on conscious experiences of self and world. More specifically, they appear to “broaden” the scope of conscious contents, vivifying imagination^8^ and positively modulating the flexibility of cognition^9^,^10^. At the same time, the states they induce are not accompanied by a global loss of consciousness or the marked changes in physiological arousal as seen in sleep or anaesthesia. These observations raise the question of whether theoretically-grounded measures of conscious level would be changed in the psychedelic state.

Empirical measures of conscious level have reached a new benchmark with the development of the perturbational complexity index, PCI^11^. The PCI quantifies the diversity across channels and observations of the EEG response to a transcranial magnetic stimulation (TMS) pulse and has been shown to robustly index levels of consciousness^6^, ranging from anaesthesia induced by various substances^11^,^12^, sleep stages^11^ and graded disorders of consciousness such as (emergence from) the minimally conscious state^11^,^13^. Notably, all these comparisons resulted in lower PCI values compared to a baseline state of wakeful awareness.

One disadvantage of the PCI approach is that it requires brain stimulation, which limits its applicability and generalisability. A complementary approach is therefore to measure signal diversity of spontaneous neural activity recorded under various manipulations of conscious level. Following early studies of anaesthetics^14^,^15^,^16^ and natural sleep states^17^,^18^, we recently found reliable reductions in neural signal diversity with diminished conscious level across a range of measures and experimental manipulations, focusing on spontaneous electrophysiological recordings. These measures include: versions of Lempel-Ziv complexity (LZc, LZs), which quantify the number of distinct patterns present in the data; amplitude coalition entropy (ACE), which reflects the entropy over time of the constitution of the set of most active channels; and synchrony coalition entropy (SCE), which reflects the entropy over time of the constitution of the sets of synchronous channels. These measures of signal diversity robustly index levels of propofol sedation^19^ and sleep stages^20^,^21^ when applied to spontaneous electrophysiological recordings. As with the PCI studies, these measures were reliably higher for conscious than for unconscious conditions.

Measures of entropy and Lempel-Ziv complexity both capture the diversity of a signal. In the limit of an infinitely long binary string, Lempel-Ziv complexity^22^ becomes directly proportional to the entropy of the process generating the string, provided the process is ergodic. Further, it can provide a good approximation to the entropy of a binary string if its length is of order of magnitude 1000 or greater^23^, a length easily obtainable for MEG/EEG data segments spanning just a few seconds. Note however, that a reordering of the components of a string can change the Lempel-Ziv complexity. For example, if all the 1 s are grouped together then the Lempel-Ziv complexity goes to approximately zero. By contrast, reordering does not affect the entropy. The Lempel-Ziv complexity and entropy measures considered here (LZc, LZs, ACE, SCE) go beyond characterising a single binary string (except LZs), e.g. for the coalition entropy measures each component in the considered string is a subset of the set of observed channels. Thus, the relations between these measures is more complicated. Indeed, these measures have been shown to diverge in their behaviour in certain scenarios^19^, such as when there is high correlation between channels. Thus, it is valuable to consider the behaviour of these measures collectively, when characterising signal diversity.

Functional MRI-based measures of entropy have previously been found to be greater in the psychedelic state than in normal waking consciousness^8^,^24^,^25^,^26^ and this effect has been related, both theoretically^8^,^24^ and empirically^8^,^26^, to the phenomenal qualities of the psychedelic state. Given that Lempel-Ziv complexity can quantify the true entropy of certain stochastic processes more accurately than direct approximate entropy measures^23^, it is arguably more sensitive to signal diversity than entropy measures that have been applied previously to psychedelic data. Moreover, no such measures have previously been applied to data derived from EEG or MEG recordings of the psychedelic state. EEG/MEG data have far higher temporal resolution than fMRI and therefore are much better suited for signal diversity analyses. In addition, using Lempel-Ziv complexity allows analyses of the psychedelic state to be compared with similar analyses applied to more global changes in conscious level, as previously described^19^,^20^,^21^.

Here, we sought to test the hypothesis that three different psychedelic drugs (psilocybin, LSD and sub-anaesthetic ketamine), known to produce unusual altered states of consciousness, characterised by rich phenomenal content, would yield scores of signal diversity exceeding those for normal waking consciousness. For parsimony, ketamine is referred to as a ‘psychedelic’, while acknowledging that its pharmacology and subjective effects are somewhat different to those of the ‘classic’ serotonergic psychedelics, such as LSD and psilocybin. We did this by re-analysing multidimensional spontaneous MEG recordings using our measures of spontaneous signal diversity. We compared signal diversity for two conditions: post-placebo and post-psychedelic drug. We further examined whether changes in measured signal diversity could be related to subjective phenomenological descriptions obtained following drug administration, in order to test whether these changes reflected specific aspects of the altered phenomenology of the psychedelic state, and to shed additional light on the complex relations linking conscious level and content.

## Methods

### Overview

We re-analysed MEG recordings from healthy subjects with open eyes, after taking a placebo and after taking a psychedelic drug. The data come from three different experiments; in each, a different drug was administered intravenously to different participants: lysergic acid diethylamide (LSD)^27^, ketamine (KET)^10^ and psilocybin (PSIL)^28^. After artefact removal and source modelling (see following sections for details), we analysed 2 *sec* segments of 90 source channels at 600 Hz: 5–7 min data for 15 participants for LSD, 6–10 min data for 19 participants for KET and 2–5 min data for 14 participants for PSIL, each time comparing resting state MEG for the drug condition with a placebo condition.

### Ethics statement

All studies were approved by a UK National Health Service research ethics committee and participants gave informed consent. Experiments were performed in accordance with relevant guidelines and regulations.

### Participants and drug dose

For all three datasets, participant exclusion criteria have been previously described in detail (PSIL^28^, KET^10^, LSD^27^). Briefly, participants were excluded if they were younger than 21, pregnant, had personal or immediate family history of psychiatric disorder, suffered from substance dependence, had cardiovascular disease, suffered from claustrophobia, blood or needle phobia, had ever had a significant adverse response to a hallucinogenic drug, or if they had a medically significant condition rendering them unsuitable for the study. All participants had previous experience with a hallucinogenic drug but not within 6 weeks of the study (for LSD and PSIL only). For KET, participants were additionally excluded if they smoked, were female, or had a body mass index outside the range of 18–30 *kg*/*m*^2^.

LSD and PSIL were each administered intravenously at a fixed single dose of 75 *μg* and 2 *mg*, respectively, over the course of less than one minute. By contrast KET was administered with an initial bolus of 0.25 *mg*/*kg* delivered over one minute followed by maintenance infusion at a rate of 0.375 *mg*/*h* for forty minutes. PSIL and KET data were obtained immediately after drug administration, whereas for LSD the data were obtained four hours after drug administration due to LSD’s slow pharmacodynamics.

### Data acquisition and preprocessing

Participants lay in a supine position for KET and LSD but were seated for PSIL. Pulse rates and blood oxygenation levels were continually monitored throughout the experiment via a probe over the left hand index finger. Whole-head MEG recordings were made using a CTF 275-channel radial gradiometer system sampled at 1200 Hz (0–300 *Hz* band-pass). An additional 29 reference channels were recorded for noise cancellation purposes and the primary sensors were analysed as synthetic third-order gradiometers. For LSD and KET, in addition to the MEG channels, ECG, horizontal and vertical participant electro-oculograms, and electromyograms from the bilateral frontalis and temporal muscles were obtained and participant compliance was monitored via an eyetracking camera.

All MEG recordings were band-pass filtered (1–150 *Hz*), downsampled to 600 *Hz* and segmented into epochs of 2 *sec* length. Each epoch was then visually inspected, and those with gross artifacts (e.g. head movements, jaw clenches) were removed from the analysis. An automated algorithm was used to remove further epochs contaminated with muscle artefacts. In this algorithm, a set of 30 gradiometer sensors were predefined at the edge of the MEG dewar (vacuum flask), as these are most likely to be contaminated by muscle artefacts. Using Hanning windowed fourier transformations, the mean spectral power for these sensors in the 105–145 *Hz* frequency band for each epoch was calculated. If the resulting power averaged across these sensors exceeded 10 *fT*^2^, then that epoch was eliminated from subsequent analysis. On the remaining epochs, independent component analysis (ICA) was performed, as implemented in Fieldtrip/EEGLAB, to identify and remove ocular, muscle and cardiac artifacts from the data. For LSD and KET, any components that showed a correlation (*r* > 0.1) in the time domain with the EOG/EMG electrodes were automatically removed, whereas these were identified manually for the PSIL data. Likewise, any components that showed correlations (*r* > 0.1) with similarly filtered EOG/EMG channels after being bandpass filtered in the range 105–145 *Hz* were removed. Visual inspection was also used to remove artifact components.

Source modelling of the data was performed using the fieldtrip toolbox^29^. For each participant, individual forward models were generated from their individual structural MRI scan^30^. In order to reduce the data, an atlas-based beamformer approach was used^31^. Broadband virtual sensor time-series were constructed using a linearly constrained minimum variance beamformer^32^ at 90 cortical and subcortical seed locations as specified in the automated anatomical labelling atlas^33^.

Prior to computing signal diversity measures, the data were further low-pass filtered with cut-off at 30 Hz to assure that possible muscle artefacts were excluded. Some residual muscle artefacts were seen in the LSD dataset^27^ and so the conservative approach was to apply the 30 Hz low-pass filter to all datasets.

### Measures

#### Lempel-Ziv complexity

**Figure 1:**
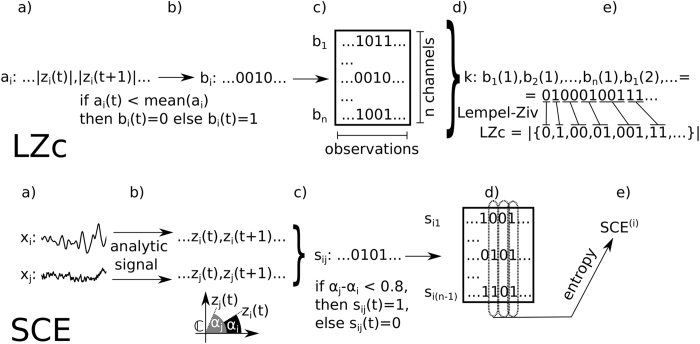
Schematic of the computation of LZc and SCE. LZc: (**a**) *x*_*i*_ is the activity of the *i*^*th*^ channel and *a*_*i*_ is the (Hilbert) amplitude of *x*_*i*_. (**b**) *b*_*i*_ is *a*_*i*_ binarised, using the mean activity of *a*_*i*_ as the binarisation threshold. (**c**) After binarisation of all *n* signals, (**d**) the multidimensional time series are concatenated observation-by-observation into one binary sequence *k* and then (**e**) repeated patterns are searched and listed into a dictionary of binary words via a Lempel-Ziv algorithm. Lempel-Ziv complexity LZc is proportional to the size of this dictionary. SCE: (**a**) Two time series. (**b**) The analytic signals of these two, which are complex signals with the real part being the original signal and the imaginary part being the Hilbert transform of the original signal. (**c**) A binary synchrony time series is created for this pair of signals; a 1 indicates that the phases of the complex values of the analytic signals are similar (difference of less than 0.8 modulo 2*π*). (**d**) Such time series are obtained to represent each channel’s synchrony with seed channel *i*. e) SCE^(*i*)^ is the entropy over observations in the resulting data matrix. The overall SCE is then the mean value of SCE^(*i*)^ across choices of seed channel *i*.

(LZc, LZs, LZc_N_, LZs_N_); We computed Lempel-Ziv complexity following our previous studies^19^,^21^. As schematically shown in Fig. 1, the instantaneous amplitude (obtained via Hilbert transform) of each source channel is binarised using its mean over observations as a threshold. I.e. the continuous signal of each source channel is transformed into a string of 1200 binary digits (for our case of 2 *sec* segments at 600 *Hz* sampling rate), resulting in a matrix with binary entries with a row for each channel and a column for each observation. To assess the signal diversity across all channels and observations, this binarised data matrix is concatenated observation-by-observation into one binary string. Then the encoding step of the Lempel-Ziv 1978 (LZ78) compression algorithm (implemented by adapting open source code) is applied to this binary string. The LZ78 algorithm divides the string into non-overlapping and unique binary substrings. The more diverse the binary string, the more substrings will be listed (a sequence containing only zeros or only ones would lead to the minimal number of substrings being obtained). The total number of these substrings is what we call Lempel-Ziv complexity LZc.

Given the observation-by-observation concatenation of the binarised data matrix, LZc captures temporal signal diversity of single channels as well as spatial signal diversity across channels. In order to assess temporal signal diversity only, we further applied this procedure to single source channels independently, and we denote the resulting single channel Lempel-Ziv complexity by LZs. I.e. LZs quantifies the temporal signal diversity of single channels, with a high score for a uniformly random binary string and a low score for a string of zeros only.

We normalize LZc (also LZs) by dividing the raw value by the value obtained for the same binary input sequence randomly shuffled. Since the value of LZc (also LZs) for a binary sequence of fixed length is maximal if the sequence is entirely random, the normalized values indicate the level of signal diversity on a scale from 0 to 1.

In order to test if changes in the diversity measures in the drug vs placebo contrast can be explained away by previously characterised changes in the overall power spectrum, we employed the following control procedure (as in ref. 19). We generate surrogate time series through phase-shuffling of the data, maximising signal diversity as measured by non-normalised LZs (analogously non-normalised LZc) under the constraint of preserving the spectral power profile of the data. If the observed difference in non-normalised LZs between drug and placebo was completely preserved in the equivalent contrast for the surrogate data, we would conclude that the observed difference is entirely due to changes of the power spectrum and not due to changes in signal diversity beyond spectral changes. If, on the other hand, the difference was completely absent for the surrogate data, we would conclude that the observed difference in non-normalised LZs was entirely due to changes in signal diversity that were not expected from the spectral power profiles.

We apply this rationale by comparing the ratio of diversity scores for data and surrogate data as follows. Assume that *D*_1_ is the LZs (LZc) score for the placebo condition (state 1) and *D*_2_ that for the drug condition (state 2). Assume *D*_1_ < *D*_2_ and let *N*_1_ be the average LZs score of the phase-shuffled data of state 1 (analogously *N*_2_ for state 2). If *D*_1_/*N*_1_ > *D*_2_/*N*_2_, then it must be that *N*_1_ < *N*_2_. Thus, this outcome implies that the difference in signal diversity between the states, *D*_1_ < *D*_2_, can be entirely explained by the difference in power spectra alone, as the maximal signal diversity given the spectral power profile of state 1 is much smaller than that of state 2. Conversely, without such reversal of results we can exclude that the observed signal diversity changes are entirely due to changes in the spectral power profile, i.e. some of the difference in diversity between the states must be due to the specific properties of the data captured by LZs (analogously LZc).

For a given participant and condition we thus computed LZc (LZs) for all 2 sec data segments. Then we phase-randomised each channel’s time series for each 2 *sec* segment and then recalculated LZc (LZs). Phase randomisation was performed by first applying a discrete Fourier transform, then randomising phases of the frequencies while keeping their amplitudes fixed, and then applying the inverse Fourier transform (i.e. only the relative temporal positions [phases] of the Fourier sinusoids, whose superposition equals the signal, are randomly changed). For a given participant and condition, measuring the average diversity across many phase-shuffled data segments gives the highest signal diversity possible for the spectral power profile for that participant in that condition, i.e. *N*_1_. We denote Lempel Ziv complexity measures normalised by average scores for phase-randomised data (*D*_1_/*N*_1_) as LZc_N_ (LZs_N_).

#### Coalition entropy

(ACE, SCE, ACE_N_, SCE_N_); Synchrony coalition entropy (SCE) is a measure of the entropy over time of the constitution of the set of channels that are in synchrony, see ref. 21 for details and Fig. 1 for a schematic. SCE normalized by its score for phase-randomised data is denoted as SCE_N_.

Amplitude coalition entropy (ACE) is defined as in ref. 21 as the entropy (over time) of the constitution of the set (coalition) of channels that are ‘active’, given the binarization scheme described above for LZc for defining ‘active’/‘inactive’ channels. As for LZc, we normalise ACE by its value for a random shuffling of the data. We further consider renormalisation by the mean value obtained from phase-randomised surrogate data (ACE_N_), as described above for LZc.

Note that ACE and SCE are only defined for multidimensional time series, capturing signal diversity over channels and time.

#### Normalized spectral power and phase coherence (PC)

The behaviour of the signal diversity measures is compared to that of normalized spectral power and phase coherence. We defined power bands as: *δ* = 1–4 *Hz, θ* = 4–8 *Hz, α* = 8–15 *Hz, β* = 15–30 *Hz* and *γ* = 30–70 *Hz*. For the computation of spectral power, the data was not low-pass filtered at 30 *Hz*, as was the case prior to the computation of the signal diversity measures. The power of a spectral band was computed using Welch’s method^34^ for each 2 *sec* segment of each of the 90 sources, normalised by the sum of the power of all 5 bands, then averaged across sources and trials per subject.

As a measure of overall synchrony we use the mean phase coherence (PC) across all pairs of channels. Let 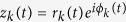 and 
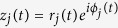
 describe the analytic signals of two channels at time *t*. Then


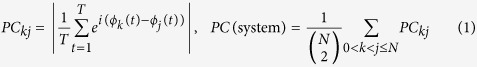


with *T* being the length of the segment (1200 observations = 2 *sec*) and *N* = 90 the number of sources. Reported PC scores are averages across trials. Scores of PC lie between 0 and 1, with 1 indicating perfect synchrony and 0 indicating no synchrony at all (phase differences uniformly randomly distributed).

### Questionnaire scores

Across the three experiments, different questionnaires were employed for participants to retrospectively evaluate their psychedelic experience. Here, we analyse a subset of the questionnaire items that were common across all three experiments, i.e. for PSIL, KET and LSD:

**strange:** “Things looked strange”.

**geom:** “I saw geometric patterns”.

**vivid:** “My imagination was extremely vivid”.

**time:** “My perception of time was distorted”.

**space:** “My sense of size and space was distorted”.

**ego:** “I experienced a disintegration of my ‘self’ or ‘ego’”.

**muddle:** “My thinking was muddled”.

**merge:** “I experienced a sense of merging with my surroundings”.

**control:** “I feared losing control of my mind”.

**spirit:** “The experience had a spiritual or mystical quality”.

**peace:** “I felt a profound inner peace”.

**float:** “I felt like I was floating”.

**past:** “I saw events from my past”.

**sounds:** “Sounds influenced things I saw”.

For all three experiments subjective questionnaires were completed retrospectively on the day of the experiments after most drug effects had subsided, asking participants to recall experiences they had at the time of peak drug effects. For KET and PSIL this was typically an hour after drug delivery had ceased but was approximately 8 hours after LSD due to its relatively prolonged pharmacodynamic profile. MEG scans were taken for KET and PSIL at peak drug effects, whereas for LSD, MEG scans were typically taken one to two hours post peak effects, i.e. immediately post drug administration for KET and PSIL, and around four hours post drug administration for LSD. Participants answered each question using a visual analogue scale format with a bottom anchor of “no, not more than usually” and a top anchor of “yes, much more than usually”. In addition, we consider the mean score over all these questions as an index of overall intensity of the psychedelic state. We call this index “total”.

Further, we obtained a single subjective rating of the overall drug-effect intensity, acquired while participants were inside the MEG scanner. This rating is for each drug denoted as “InScanner” and was obtained for LSD^27^ and PSIL^28^ by asking participants “please rate the intensity of the drug effects during the last scan” and for KET^10^ by asking “please rate your subjective high on a scale between 0 and 40”, using a two-digit button box.

### Statistics

Analyses were performed using non-overlapping segments of length 2 *sec* for a total length between 2 *min* and 10 *min* of MEG recording per participant and state. For each segment, the signal diversity measures ACE, SCE and LZc were computed for 30 random picks of 10 channels, and the mean across these 30 scores was considered the score for the segment. We chose 10 channels since in our previous study^19^ this was the smallest channel number for which we still found a robust decrease of ACE, SCE and LZc for EEG signals in propofol-anaesthesia. To verify that the results obtained were not dependent on the particular random channel selection, we performed a re-run of the complete analysis and indeed obtained almost identical results. For LZs, the mean across all 90 channels was set as the score for the segment. The mean and standard error of the diversity measures’ scores were computed across segments. At the single participant level, the effect size of differences between states was measured using Cohen’s *d*^35^. We call an effect size high if *d* > 0.7. For group level comparisons, a two-sided t-test was applied, with Bonferroni correction (by the number of measures) where indicated.

### Computation of correlation between measures

We computed the Pearson correlation across participants for the score differences of 14 measures (measure(drug)-measure(placebo)): The diversity measures ACE, LZs, LZc and SCE, their phase-randomised renormalised versions ACE_N_, LZc_N_, LZs_N_, SCE_N_, phase coherence and normalised spectral power in the delta, theta, alpha, beta and gamma band. For a given participant, trial and measure, we subtracted this measure’s score for the placebo condition from that of the drug condition to obtain a score difference for one trial. The average value across trials was used for this measure and participant. The Pearson correlation *r* is then computed for such scores for two measures across all participants for a given drug.

## Results

### Increased spontaneous signal diversity for all three drugs

Our main question was whether spatio-temporal signal diversity of MEG recordings increases in the psychedelic state, so we computed LZs, LZc, ACE and SCE for all three drug and placebo conditions and compared their scores on the participant and group level. To exclude the possibility that any observed changes could be attributed solely to changes in overall spectral profile, we also computed these diversity measures normalised by their scores for phase-shuffled data, denoted by subscript “N”: ACE_N_, LZc_N_, LZs_N_ and SCE_N_.

**Figure 2:**
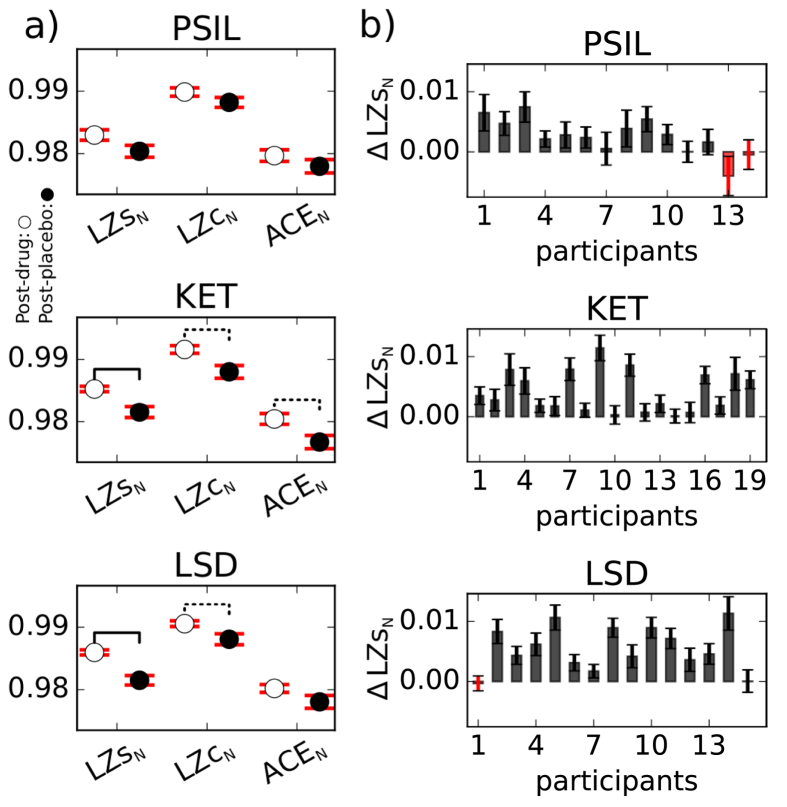
Increased spontaneous signal diversity for PSIL, KET and LSD. (**a**) Mean scores across participants for the signal diversity measures LZs_N_, LZc_N_ and ACE_N_ are higher for each of the three drug conditions (white discs) than for the corresponding placebo conditions (black discs). A solid line across conditions indicates *p* < 0.001 and a dotted line 0.001 < *p* < 0.05, uncorrected, obtained from a two-sided t-test. (**b**) Difference in single channel Lempel-Ziv complexity, LZs_N_, between respectively PSIL, KET, LSD and placebo at the single participant level. The error bars indicate standard error across trials.

Across all drugs and all diversity measures (except SCE_N_), we found increased signal diversity in the psychedelic state as compared to the placebo condition, with most comparisons reaching statistical significance, see Table 1. The most consistent increase for all three drugs when compared to placebo, was for LZs_N_, with higher average scores for 86%, 100% and 93% of participants for PSIL, KET and LSD respectively (Fig. 2b), resulting in higher LZs_N_ at the group level with *p* < 0.05 (Bonferroni corrected) for KET and LSD. Notably, the two participants for whom a higher score for PSIL was not observed also had the lowest average score across all subjective ratings for the intensity of the psychedelic experience (“total”) among all participants (see also Sections “Questionnaire scores” and “Neurophenomenological correlations”). Considering other measures, notably ACE, LZc and LZs, all scored on average significantly higher for both KET and LSD than placebo (*p* < 0.05, Bonferroni corrected). The differences for PSIL versus placebo didn’t reach significance for any of the measures, although a higher value for PSIL was observed for most subjects. Figure 2a shows the results for the measures ACE_N_, LZc_N_, LZs_N_ at the group level. The fact that single channel Lempel-Ziv complexity, as captured by LZs_N_, exhibited the most strongly significant increase of the diversity measures suggests that increased temporal signal diversity is a stronger hallmark of the psychedelic state than spatial signal diversity.

That in addition to ACE, LZc and LZs also the phase-randomisation renormalised measures ACE_N_, LZc_N_ and LZs_N_ all exhibit higher values for the clear majority of subjects for each of the drugs in comparison to placebo (and significantly so in several cases) illustrates that there is a generalised increase in signal diversity in the psychedelic states beyond that expected from spectral changes (see “Methods” for details of this control). However, as effect sizes at the single participant level were generally lower for the phase-randomisation renormalised measures, there was some increase in signal diversity deriving from spectral changes (Table 1).

For comparison with the signal diversity measures, we computed phase coherence and normalized spectral power for various frequency bands, for all three drugs and the placebo condition. All 90 sources were used to compute these measures (Section “Normalized spectral power and phase coherence (PC)”). Strongest changes in the average power spectrum were seen for the alpha band (8–15 *Hz*), where a decrease in power was observed for all drugs relative to placebo, with large effect sizes for the majority of participants (in line with refs 10, 27, 28), see Table 1. For phase coherence (PC), we found no significant difference between any drug and placebo.

**Figure 3:**
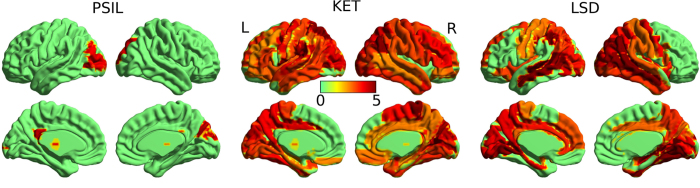
t-statistics across participants of ΔLZs_N_ per source channel. Group level changes in single channel Lempel-Ziv complexity, ΔLZs_N_, are indicated by the t-statistic for each of the 90 source channels and 3 drugs (where significant at the *p* < 0.05 level, FDR corrected). For all three drugs, an increase in LZs_N_ can be seen in occipital-parietal areas. Image created using BrainNet viewer^55^.

To examine whether changes in signal diversity showed any anatomical localisation, we compared local signal diversity changes across drugs, using phase-randomisation renormalised single-channel Lempel-Ziv complexity LZs_N_. For a given drug, participant and source channel, ΔLZs_N_ = LZs_N_(drug) − LZs_N_(placebo) was obtained as an average across all 2 *sec* data segments. For each of the 90 sources, the t-statistic obtained for the ΔLZs_N_ scores across participants was mapped in color onto a standard MNI brain, shown in Fig. 3 (wherever significant at the *p* < 0.05 level, false discovery rate [FDR] corrected). For all three drugs, substantial increases in LZs_N_ can be seen in occipital-parietal areas, despite differences in pharmacological target region and psychological effects for each drug. The regions with significant changes for PSIL also had significant changes for KET and LSD, and are thus the regions with significant changes across all drugs. These spatial distributions, with maximal locations in occipital and parietal areas are consistent with the localisation of alpha-band changes that were previously reported^10^,^27^,^28^.

In summary, we found a clear increase in signal diversity for all three psychedelic agents at the group level, with effects being strongest for KET. These increases went beyond those expected from the changes to the frequency spectrum. We further confirm a consistent decrease in normalised alpha power for all three drugs, in line with previous analyses of the data^10^,^27^,^28^.

### Correlations between neurophysiological measures

**Figure 4:**
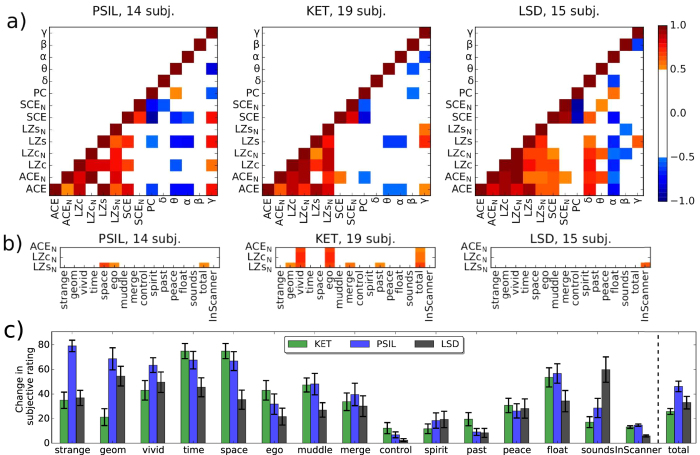
Correlations across measures and questionnaire answers. (**a**) For each drug, a matrix indicates in colour the Pearson correlation, *r*, of the score difference between drug and placebo condition (averaged across trials) of each measure pair across participants. The upper triangular entries and entries with |*r*| < 0.5 are omitted (and set white) to highlight strong correlations only. These correlations should be considered somewhat exploratory and are not controlled for multiple comparisons as each experiment had a limited sample size. Across drugs, signal diversity measures show high correlation with each other, yet inconsistently, as they capture different flavours of signal diversity. (**b**) Correlations between the changes in the measures ACE_N_, LZc_N_ and LZs_N_ and the questionnaire scores (same scale as above). No consistent correlation across drugs was found for any combination of the measure’s scores and scores for a particular question. (**c**) The changes in subjective ratings under each drug condition are shown as averages with standard error bars across subjects. The average of all changes across these 14 questions (except “InScanner”) is denoted as “total” and shown normalised by factor 20 in order to fit the scale. No consistent differences are apparent across drugs.

To test whether the various measures (both signal diversity measures and others) were reflecting distinct features of the data, we computed correlations between the changes in these measures, across participants, for each comparison between drug and placebo. The Pearson correlation *r* is computed for two measures across all participants for a given drug (see “Methods” for details) and indicated in colour in Fig. 4 if *r* > 0.5, in order to highlight moderate and stronger correlations only. For clarity, the upper triangular portion of the symmetric correlation matrices is hidden.

We found strong correlations across most diversity measures for all three drugs. Slight inconsistencies in these correlations across drugs show that the measures capture not identical signal features, in line with their varying behaviour as listed in Table 1 and their distinct mathematical definitions. Most diversity measures show stronger correlations with normalised spectral power bands when compared to their versions normalised by their scores for phase-randomised data (indicated by subscript N), verifying that the phase-shuffling normalisation indeed reduced the measures’ sensitivity to spectral changes, as intended. Phase coherence, PC, did not correlate strongly with the diversity measures; in particular, correlations between PC and LZs_N_ were weak. (See Fig. S1 for full correlation matrix).

### Neurophenomenological correlations

Having established that signal diversity measures increase in the psychedelic state, we next asked whether these increases were related to subjective measures. We therefore computed the Pearson correlation for all combinations of the measures LZs_N_, LZc_N_ and ACE_N_ with the scores of 14 subjective ratings of the participants’ psychedelic experience acquired after the drug effects had faded, i.e. post-experiment, and one rating obtained in the MEG scanner (“InScanner”) at a time when the drug effects were still strong, as defined in Section “Questionnaire scores”. The results are shown in Fig. 4b. (See Fig. S1 for correlations with the entire list of measures).

Supporting a relation between phenomenology and signal diversity, the measure LZs_N_, which showed the strongest overall response to the psychedelic state across drugs, correlates substantially (*r* > 0.5) with the total score for all questions (“total”) for PSIL and KET. Recall that the two participants with the lowest total score across all questions for PSIL were the two outlying participants for ΔLZs_N_ as shown in Fig. 2b (i.e. these participants did not show an increase in LZs_N_ under PSIL). These observations support the notion that LZs_N_ correlates with the intensity of the psychedelic experience.

In addition, strong correlations were found between all 3 signal diversity measures and the total score across questions for KET. For this drug, all three diversity measures also showed further strong correlations with specific phenomenological dimensions, in particular ego dissolution and vivid imagination. However, for LSD only “InScanner” correlated strongly with LZs_N_ while for PSIL, LZs_N_ correlated strongly with scores for only two of the individual questions in addition to the total score. The absence of strong correlations between post-experiment subjective ratings and signal diversity for LSD may be due to the fact that in the LSD study, the questions were asked with reference to the period when subjective effects were maximal and not for the time in the MEG scanner (2 h post drug effect peak), unlike for KET and PSIL, where the MEG scan took place close to drug effect peak. However, no strong neurophenomenological correlations involving “InScanner” ratings were found for PSIL and KET. For certain phenomenological dimensions, such as “space” and “ego” which correlated with LZs_N_ for PSIL, the subjective ratings were weaker for LSD in comparison to the other drugs, although this was not the case for all dimensions and, importantly, for the scores of “total” difference in phenomenology, for LSD lay above that for KET and below that for PSIL, suggesting that the overall intensity of the psychedelic experience induced by LSD was comparable to the other drugs, see Fig. 4c.

In sum, subjective ratings of the psychedelic experience correlate most strongly with spontaneous signal diversity for KET, and while some strong correlations exist also for PSIL, the only strong neurophenomenological correlation observed for LSD involved “InScanner” ratings. The total score across all questions, being an index of the overall intensity of the psychedelic experience, correlates strongly with LZs_N_ for PSIL and KET. Given the constraints on the acquisition of subjective reports, these findings tentatively support a relationship between psychedelic phenomenology and neural signal diversity as discussed below.

## Discussion

We have demonstrated, for the first time, that measures of neural signal diversity that are known to be sensitive to conscious level, are also sensitive to the changes in brain dynamics associated with the psychedelic state. We found that the psychedelic state induces increased brain-wide signal diversity as compared to placebo, across a range of measures and three different psychedelic compounds. The measures LZc, LZc_N_, LZs, LZs_N_, ACE and ACE_N_ all scored higher at the group level for the drugs PSIL, KET and LSD, with strongly significant increases, consistent across subjects, seen for ACE, LZc, LZs and LZs_N_ for KET and LSD. Importantly, by utilising phase-shuffled surrogate data, we excluded the possibility that the observed increases in signal diversity could be explained away by changes in the spectral profile induced by the drugs. Together, these findings constitute a new neural correlate of the psychedelic state that may have important broader implications for our understanding of the neural correlates of consciousness.

Despite the differing pharmacological mechanism of action of KET, LSD and PSIL, we observed a clear similarity in the cortical localisation of changes in signal diversity measures - with relatively overlapping spatial distributions centered over occipital and parietal cortices. These areas are strikingly similar to the locations of alpha power decreases that we have previously reported^10^,^27^,^28^, yet spectral changes alone do not account for changes in signal diversity as our “phase-randomisation” control showed. The primary psychedelic effects of LSD and PSIL are thought to be mediated via 5HT2A receptors^36^,^37^, which although distributed throughout the neocortex, have somewhat higher levels in occipital-parietal areas^38^,^39^. Conversely, KET’s primary mechanism of action is as an NMDA antagonist whose receptors are located quite ubiquitously across the cerebral cortex as well as subcortically^40^,^41^. One could speculate that some of the shared phenomenological and electrophysiological effects of these drugs may be mediated by the known interactions between 5HT2A receptors and NMDA receptors^42^,^43^. Alternatively, 5HT2A receptor agonism and NMDA receptor antagonism may have similar effects on the activity of cortical cell populations^44^,^45^. This could account for the localised and overlapping areas of both signal diversity and decreased alpha power that we have observed with all three drugs. However, the non-NMDA receptor effects of KET cannot be discounted, in particular its interactions with opioid receptors and HCN channels^46^,^47^. Alternatively, it may simply be that MEG/EEG is well tuned to measure changes in these cortical areas due to local synchronisation properties of the underlying circuits in these areas.

Neural correlates of consciousness are particularly valuable when they can be mapped to phenomenological properties^48^,^49^. For KET, strong correlations across participants were found between scores of LZc_N_, LZs_N_ and ACE_N_ and specific subjective ratings of the psychedelic experience, in particular for ego-dissolution and vividness of the experience, as well as the total score across all questions - reflecting the overall intensity of the psychedelic experience. For PSIL, LZs_N_ showed strong correlation with three questions, including the total “intensity” score, while for LSD, strong correlations were only found between signal diversity and the subjective ratings acquired in the scanner but not for ratings acquired at a time when the drug effects had faded. The weaker correlations found for LSD are likely to be due to the fact that participants were asked to rate their experience during peak drug effects, which was 2 h prior to the MEG scan, while for KET and PSIL the MEG scan took place when drug effects were at peak. Nevertheless, for the scores of “total” difference in phenomenology, LSD lay above KET and below PSIL, suggesting that the overall intensity of the psychedelic experience induced by LSD was not lower than for the other drugs.

While we^10^,^27^,^28^ and others^50^ have used the methodological approach of correlating subjective states with electrophysiological measures across participants, it should be noted that this is an imperfect approach. In particular, the ratings of any individual are heavily influenced by their individual biases and histories - each participant has their own yardstick for evaluating the strength of an experience. Further, the retrospective nature of the ratings relies on recall of the experience as a single entity and does not capture the dynamic, periodic features of the psychedelic experience. Future experiments which seek to capture temporal variation in experience through use of multiple probe items, experience sampling methods or perhaps even by spontaneous self-report, and retrospective coding, may help to more tightly tie neurophysiological measures such as those we have used here to subjective experiences.

Correlations of perturbational and spontaneous signal diversity with conscious states support integrated information and complexity theories of consciousness that emphasise diversity of phenomenology as a key property of consciousness that must be reflected in its neural correlates^1^,^48^,^51^,^52^,^53^. Perturbational^11^,^12^ and - with weaker specificity and sensitivity - spontaneous signal diversity measures capture types of neural signal diversity - across broadly distributed brain regions - that correlate with changes in conscious level across a broad range of states. Typically, research has focused on manipulations or pathologies involving a diminution of the overall level of consciousness from a baseline of healthy conscious wakeful rest^19^,^21^. Pragmatically, these results suggest an operationally useful one-dimensional scale for level of consciousness, with wakeful rest and REM sleep at the top and coma and propofol-induced general aneasthesia at the bottom. Our findings of *increased* spontaneous signal diversity for KET, PSIL and LSD presented here, represent the first observations of an increase in theoretically-motivated measures of conscious level with respect to the baseline of wakeful rest, on such a one-dimensional scale. These results broaden the scope of application of signal diversity measures relevant to conscious level, showing that the one-dimensional scale extends in both directions from the baseline state. While it may be tempting to describe the psychedelic state as a “higher” state or level of consciousness on the basis of our findings, any such description needs to be cautiously interpreted and properly qualified.

The measures applied in this paper focus on signal diversity, rather than the simultaneous existence of integration and differentiation (or integrated information) that are emphasised within complexity and integrated information theories of consciousness^48^. Our diversity measures are attractive because of simplicity, practical applicability, and consistency with both complexity-based^48^ and entropy-based^8^ theories about consciousness, providing a quantitatively useful mapping between neural signal dynamics and phenomenology.

Our findings of reliable changes in signal diversity in the psychedelic state suggest that further research could usefully consider less common alterations of consciousness, for example manic, dreamlike, delirious conditions. In these conditions, as in the psychedelic state, conscious scenes may be “richer”, or more “expansive” or “diverse” than normal.

Our results provide an example of how quantitative measures of neural dynamics can bridge the gap between studies of conscious content and conscious level and are in line with the intuitive suggestion that increases in conscious level correspond to increases in the range of possible conscious contents^2^,^3^,^8^. Recently, efforts to finesse the relationship between level and content have been made in the context of integrated information theory^54^ and in multidimensional descriptions of conscious level^2^. Interestingly, Bayne *et al*. suggest a multidimensional classification of conscious levels, with one dimension being the depth of context an observer grasps on average: for example whether only the colour of an object is perceived, or whether its purpose is also perceived. Distinctions like this may be useful in characterising the phenomenology of the psychedelic state. Further research into the relation between phenomenology and the detailed expression of measures of complexity and diversity at local and global levels will help refine and constrain these emerging ideas.

In sum, we found increased global neural signal diversity for the psychedelic state induced by KET, PSIL and LSD, suggesting the psychedelic state lies above conscious states such as wakeful rest and REM sleep on a one-dimensional scale defined by neural signal diversity. Future studies should assess the extent to which entropy and complexity based measures of signal diversity capture and confer the fundamental property of “richness” of conscious states, not only in the psychedelic condition but in conscious states more generally.

## Additional Information

**How to cite this article**: Schartner, M. M. *et al*. Increased spontaneous MEG signal diversity for psychoactive doses of ketamine, LSD and psilocybin. *Sci. Rep.* **7**, 46421; doi: 10.1038/srep46421 (2017).

**Publisher's note:** Springer Nature remains neutral with regard to jurisdictional claims in published maps and institutional affiliations.

## Supplementary Material

Supplementary Figures

## Figures and Tables

**Table 1:** Characterisation of the changes in signal diversity, phase coherence, and power spectrum in the psychedelic state.

Measure	PSIL (*n* = 14)						KET (*n* = 19)						LSD (*n* = 15)					
	%	*t*	*p*	effect size %			%	*t*	*p*	effect size %			%	*t*	*p*	effect size %		
ACE	↑ 86	1.4	0.185	43	50	7	↑ 95	3.9	*0.000*	68	32	0	↑ 93	4.3	*0.000*	87	13	0
ACE_N_	↑ 71	0.8	0.428	0	100	0	↑ 89	2.8	**0.008**	0	100	0	↑ 73	1.7	0.093	0	100	0
LZc	↑ 79	1.5	0.154	14	86	0	↑ 100	3.7	*0.001*	37	63	0	↑ 87	3.4	*0.002*	40	60	0
LZc_N_	↑ 86	1.3	0.197	0	100	0	↑ 100	3.4	*0.002*	0	100	0	↑ 80	2.5	**0.018**	0	100	0
LZs	↑ 86	1.5	0.134	57	36	7	↑ 89	3.4	*0.002*	74	26	0	↑ 100	4.9	*0.000*	93	7	0
LZs_N_	↑ 86	1.4	0.173	0	100	0	↑ 100	3.5	*0.001*	0	100	0	↑ 93	4.8	*0.000*	0	100	0
SCE	↑ 71	0.3	0.767	36	50	14	↑ 89	2.4	**0.021**	68	26	5	↑ 60	1.1	0.293	40	40	20
SCE_N_	↓ 64	0.9	0.370	0	100	0	↑ 53	0.0	0.971	0	95	5	↓ 73	2.4	**0.022**	0	100	0
PC	↑ 64	0.7	0.509	29	71	0	↓ 74	0.9	0.365	47	47	5	↑ 67	1.1	0.300	47	33	20
*δ*	↑ 71	0.9	0.363	14	79	7	↓ 74	2.1	**0.043**	47	53	0	↓ 73	1.4	0.184	40	53	7
*θ*	↑ 64	0.5	0.636	29	57	14	↑ 68	1.4	0.159	53	37	11	↓ 80	2.2	**0.038**	73	27	0
*α*	↓ 86	2.2	**0.041**	64	29	7	↓ 95	3.2	*0.003*	79	16	5	↓ 93	3.3	*0.003*	80	20	0
*β*	↓ 86	0.7	0.519	36	57	7	↓ 79	2.0	0.057	42	53	5	↓ 67	1.0	0.339	53	33	13

For each measure and drug, the percentage of participants for which the measure was higher (indicated by up-arrow), or lower (indicated by down-arrow) in the drug condition than placebo is given, followed by the t-statistic and uncorrected *p*-value of a two-sided t-test across participants for conditions placebo and drug. The number triplet in column “effect size %” lists for which percentage of participants the measure’s score changed in the arrow-indicated direction with high effect size (left digit), in the opposite direction with high effect size (right digit) or in either direction but with low effect size (middle digit). Effect size is said to be high if Cohen’s d was greater than 0.7. The font of a *p*-value entry is in italics if the *p*-value was below 0.05 (but not the Bonferroni corrected p-value). If the Bonferroni corrected *p*-value was below 0.05, the entry is in boldface.

## References

[b1] Tononi G. Consciousness as integrated information: a provisional manifesto. The Biological Bulletin 215, 216–242 (2008). 19098144 10.2307/25470707

[b2] Bayne T., Hohwy J. & Owen A. M. Are there levels of consciousness? Trends in cognitive sciences 20, 405–413 (2016). 27101880 10.1016/j.tics.2016.03.009

[b3] Boly M. . Consciousness in humans and non-human animals: recent advances and future directions. Frontiers in psychology 4, 625 (2013). 24198791 10.3389/fpsyg.2013.00625 PMC3814086

[b4] Hohwy J. The neural correlates of consciousness: new experimental approaches needed? Consciousness and cognition 18, 428–438 (2009). 19345590 10.1016/j.concog.2009.02.006

[b5] Overgaard M. & Overgaard R. Neural correlates of contents and levels of consciousness. Frontiers in psychology 1, 164 (2010). 21887148 10.3389/fpsyg.2010.00164 PMC3157935

[b6] Koch C., Massimini M., Boly M. & Tononi G. Neural correlates of consciousness: progress and problems. Nature Reviews Neuroscience 17, 307–321 (2016). 27094080 10.1038/nrn.2016.22

[b7] Seth A. K., Dienes Z., Cleeremans A., Overgaard M. & Pessoa L. Measuring consciousness: relating behavioural and neurophysiological approaches. Trends in cognitive sciences 12, 314–321 (2008). 18606562 10.1016/j.tics.2008.04.008 PMC2767381

[b8] Carhart-Harris R. L. . The entropic brain: a theory of conscious states informed by neuroimaging research with psychedelic drugs. Frontiers in Human Neuroscience 8, 20 (2014). 24550805 10.3389/fnhum.2014.00020 PMC3909994

[b9] Carhart-Harris R. . The paradoxical psychological effects of lysergic acid diethylamide (lsd). Psychological medicine 46, 1379–1390 (2016). 26847689 10.1017/S0033291715002901

[b10] Muthukumaraswamy S. D. . Evidence that subanesthetic doses of ketamine cause sustained disruptions of nmda and ampa-mediated frontoparietal connectivity in humans. The Journal of Neuroscience 35, 11694–11706 (2015). 26290246 10.1523/JNEUROSCI.0903-15.2015 PMC4540803

[b11] Casali A. G. . A theoretically based index of consciousness independent of sensory processing and behavior. Science translational medicine 5, 198ra105–198ra105 (2013). 10.1126/scitranslmed.3006294 23946194

[b12] Sarasso S. . Consciousness and complexity during unresponsiveness induced by propofol, xenon, and ketamine. Current Biology 25, 3099–3105 (2015). 26752078 10.1016/j.cub.2015.10.014

[b13] Sarà M. & Pistoia F. Complexity loss in physiological time series of patients in a vegetative state. Nonlinear dynamics, psychology, and life sciences 14, 1 (2010). 20021774

[b14] Zhang X.-S., Roy R. J. & Jensen E. W. Eeg complexity as a measure of depth of anesthesia for patients. IEEE Transactions on Biomedical Engineering 48, 1424–1433 (2001). 11759923 10.1109/10.966601

[b15] Ferenets R. . Comparison of entropy and complexity measures for the assessment of depth of sedation. IEEE Transactions on Biomedical Engineering 53, 1067–1077 (2006). 16761834 10.1109/TBME.2006.873543

[b16] Ferenets R., Vanluchene A., Lipping T., Heyse B. & Struys M. M. Behavior of entropy/complexity measures of the electroencephalogram during propofol-induced sedationdose-dependent effects of remifentanil. The Journal of the American Society of Anesthesiologists 106, 696–706 (2007). 10.1097/01.anes.0000264790.07231.2d 17413907

[b17] Liu Z. & Sun J. Sleep staging from the eeg signal using multifractal detrended fluctuation analysis. In 2015 Fifth International Conference on Instrumentation and Measurement, Computer, Communication and Control (IMCCC), 63–68 (IEEE, 2015).

[b18] Burioka N. . Approximate entropy in the electroencephalogram during wake and sleep. Clinical EEG and neuroscience 36, 21–24 (2005). 15683194 10.1177/155005940503600106 PMC2563806

[b19] Schartner M. . Complexity of multi-dimensional spontaneous eeg decreases during propofol induced general anaesthesia. PloS one 10, e0133532 (2015). 26252378 10.1371/journal.pone.0133532 PMC4529106

[b20] Andrillon T., Poulsen A. T., Hansen L. K., Léger D. & Kouider S. Neural markers of responsiveness to the environment in human sleep. The Journal of Neuroscience 36, 6583–6596 (2016). 27307244 10.1523/JNEUROSCI.0902-16.2016 PMC6601917

[b21] Schartner M. M. . Global and local complexity of intracranial eeg decreases during nrem sleep. Neuroscience of Consciousness 3(1)niw022 (2017). 10.1093/nc/niw022 PMC6007155 30042832

[b22] Lempel A. & Ziv J. On the complexity of finite sequences. IEEE Transactions on information theory 22, 75–81 (1976).

[b23] Amigó J. M., Szczepański J., Wajnryb E. & Sanchez-Vives M. V. Estimating the entropy rate of spike trains via lempel-ziv complexity. Neural Computation 16, 717–736 (2004). 15025827 10.1162/089976604322860677

[b24] Carhart-Harris R., Leech R., Tagliazucchi E. . How do hallucinogens work on the brain? Journal of Psychophysiology 71, 2–8 (2014).

[b25] Tagliazucchi E., Carhart-Harris R., Leech R., Nutt D. & Chialvo D. R. Enhanced repertoire of brain dynamical states during the psychedelic experience. Human brain mapping 35, 5442–5456 (2014). 24989126 10.1002/hbm.22562 PMC6869695

[b26] Lebedev A. . Lsd-induced entropic brain activity predicts subsequent personality change. Human brain mapping 37(9), 3203–13 (2016). 27151536 10.1002/hbm.23234 PMC6867426

[b27] Carhart-Harris R. L. . Neural correlates of the lsd experience revealed by multimodal neuroimaging. Proceedings of the National Academy of Sciences 113, 4853–4858 (2016). 10.1073/pnas.1518377113 PMC4855588 27071089

[b28] Muthukumaraswamy S. D. . Broadband cortical desynchronization underlies the human psychedelic state. The Journal of Neuroscience 33, 15171–15183 (2013). 24048847 10.1523/JNEUROSCI.2063-13.2013 PMC6618409

[b29] Oostenveld R., Fries P., Maris E. & Schoffelen J.-M. Fieldtrip: open source software for advanced analysis of meg, eeg, and invasive electrophysiological data. Computational intelligence and neuroscience 2011 (2010). 10.1155/2011/156869 PMC3021840 21253357

[b30] Nolte G. The magnetic lead field theorem in the quasi-static approximation and its use for magnetoencephalography forward calculation in realistic volume conductors. Physics in medicine and biology 48, 3637 (2003). 14680264 10.1088/0031-9155/48/22/002

[b31] Hillebrand A., Barnes G. R., Bosboom J. L., Berendse H. W. & Stam C. J. Frequency-dependent functional connectivity within resting-state networks: an atlas-based meg beamformer solution. Neuroimage 59, 3909–3921 (2012). 22122866 10.1016/j.neuroimage.2011.11.005 PMC3382730

[b32] Van Veen B. D., Van Drongelen W., Yuchtman M. & Suzuki A. Localization of brain electrical activity via linearly constrained minimum variance spatial filtering. IEEE Transactions on biomedical engineering 44, 867–880 (1997). 9282479 10.1109/10.623056

[b33] Tzourio-Mazoyer N. . Automated anatomical labeling of activations in spm using a macroscopic anatomical parcellation of the mni mri single-subject brain. Neuroimage 15, 273–289 (2002). 11771995 10.1006/nimg.2001.0978

[b34] Welch P. D. The use of fast fourier transform for the estimation of power spectra: A method based on time averaging over short, modified periodograms. IEEE Transactions on audio and electroacoustics 15, 70–73 (1967).

[b35] Cohen J. A power primer. Psychological bulletin 112, 155 (1992). 19565683 10.1037//0033-2909.112.1.155

[b36] Halberstadt A. L. Recent advances in the neuropsychopharmacology of serotonergic hallucinogens. Behavioural brain research 277, 99–120 (2015). 25036425 10.1016/j.bbr.2014.07.016 PMC4642895

[b37] Gonzalez-Maeso J. . Hallucinogens recruit specific cortical 5-ht 2a receptor-mediated signaling pathways to affect behavior. Neuron 53, 439–452 (2007). 17270739 10.1016/j.neuron.2007.01.008

[b38] Erritzoe D. . Brain serotonin 2a receptor binding: relations to body mass index, tobacco and alcohol use. Neuroimage 46, 23–30 (2009). 19457377 10.1016/j.neuroimage.2009.01.050

[b39] Beliveau V. . A high-resolution *in vivo* atlas of the human brain’s serotonin system. Journal of Neuroscience 37, 120–128 (2017). 28053035 10.1523/JNEUROSCI.2830-16.2016 PMC5214625

[b40] Conti F., Minelli A., Molnar M. & Brecha N. C. Cellular localization and laminar distribution of nmdar1 mrna in the rat cerebral cortex. Journal of Comparative Neurology 343, 554–565 (1994). 8034787 10.1002/cne.903430406

[b41] Huntley G. W. . Distribution and synaptic localization of immunocytochemically identified nmda receptor subunit proteins in sensory-motor and visual cortices of monkey and human. The Journal of neuroscience 14, 3603–3619 (1994). 8207475 10.1523/JNEUROSCI.14-06-03603.1994 PMC6576922

[b42] Farber N. B., Hanslick J., Kirby C., McWilliams L. & Olney J. W. Serotonergic agents that activate 5ht2a receptors prevent nmda antagonist neurotoxicity. Neuropsychopharmacology 18, 57–62 (1998). 9408919 10.1016/S0893-133X(97)00127-9

[b43] Arvanov V. L., Liang X., Russo A. & Wang R. Y. Lsd and dob: interaction with 5-ht2a receptors to inhibit nmda receptor-mediated transmission in the rat prefrontal cortex. European Journal of Neuroscience 11, 3064–3072 (1999). 10510170 10.1046/j.1460-9568.1999.00726.x

[b44] Celada P. . Disruption of thalamocortical activity in schizophrenia models: relevance to antipsychotic drug action. International Journal of Neuropsychopharmacology 16, 2145–2163 (2013). 23809188 10.1017/S1461145713000643

[b45] Wood J., Kim Y. & Moghaddam B. Disruption of prefrontal cortex large scale neuronal activity by different classes of psychotomimetic drugs. The Journal of Neuroscience 32, 3022–3031 (2012). 22378875 10.1523/JNEUROSCI.6377-11.2012 PMC3531997

[b46] Chen X., Shu S. & Bayliss D. A. Hcn1 channel subunits are a molecular substrate for hypnotic actions of ketamine. The Journal of Neuroscience 29, 600–609 (2009). 19158287 10.1523/JNEUROSCI.3481-08.2009 PMC2744993

[b47] Zhou C. . Forebrain hcn1 channels contribute to hypnotic actions of ketamine. The Journal of the American Society of Anesthesiologists 118, 785–795 (2013). 10.1097/ALN.0b013e318287b7c8 PMC3605219 23377220

[b48] Tononi G. & Edelman G. M. Consciousness and complexity. science 282, 1846–1851 (1998). 9836628 10.1126/science.282.5395.1846

[b49] Seth A. Explanatory correlates of consciousness: theoretical and computational challenges. Cognitive Computation 1, 50–63 (2009).

[b50] Kometer M., Pokorny T., Seifritz E. & Volleinweider F. X. Psilocybin-induced spiritual experiences and insightfulness are associated with synchronization of neuronal oscillations. Psychopharmacology 232, 3663–3676 (2015). 26231498 10.1007/s00213-015-4026-7

[b51] Tononi G., Sporns O. & Edelman G. M. A measure for brain complexity: relating functional segregation and integration in the nervous system. Proceedings of the National Academy of Sciences 91, 5033–5037 (1994). 10.1073/pnas.91.11.5033 PMC43925 8197179

[b52] Seth A. K., Izhikevich E., Reeke G. N. & Edelman G. M. Theories and measures of consciousness: an extended framework. Proceedings of the National Academy of Sciences 103, 10799–10804 (2006). 10.1073/pnas.0604347103 PMC1487169 16818879

[b53] Tononi G. & Koch C. Consciousness: here, there and everywhere? Phil. Trans. R. Soc. B 370, 20140167 (2015). 25823865 10.1098/rstb.2014.0167 PMC4387509

[b54] Gallimore A. R. Restructuring consciousness–the psychedelic state in light of integrated information theory. Frontiers in human neuroscience 9 (2015). 10.3389/fnhum.2015.00346 PMC4464176 26124719

[b55] Xia M., Wang J. & He Y. Brainnet viewer: a network visualization tool for human brain connectomics. PloS one 8, e68910 (2013). 23861951 10.1371/journal.pone.0068910 PMC3701683

