# Supplementary material for: Increased spontaneous MEG signal diversity for psychoactive doses of ketamine, LSD and psilocybin
Source: Sci Rep. 2017 Apr 19;7:46421. doi: 10.1038/srep46421 (PMC5396066; doi:10.1038/srep46421)
Supplement: Supplementary Figures [file srep46421-s1.pdf]

Supplementary figures for:

Increased spontaneous MEG signal diversity for psychoactive doses of ketamine, LSD and psilocybin

Michael M. Schartner, Robin Carhart-Harris, Adam B. Barrett,
Anil K. Seth, Suresh D. Muthukumaraswamy

January 19, 2017

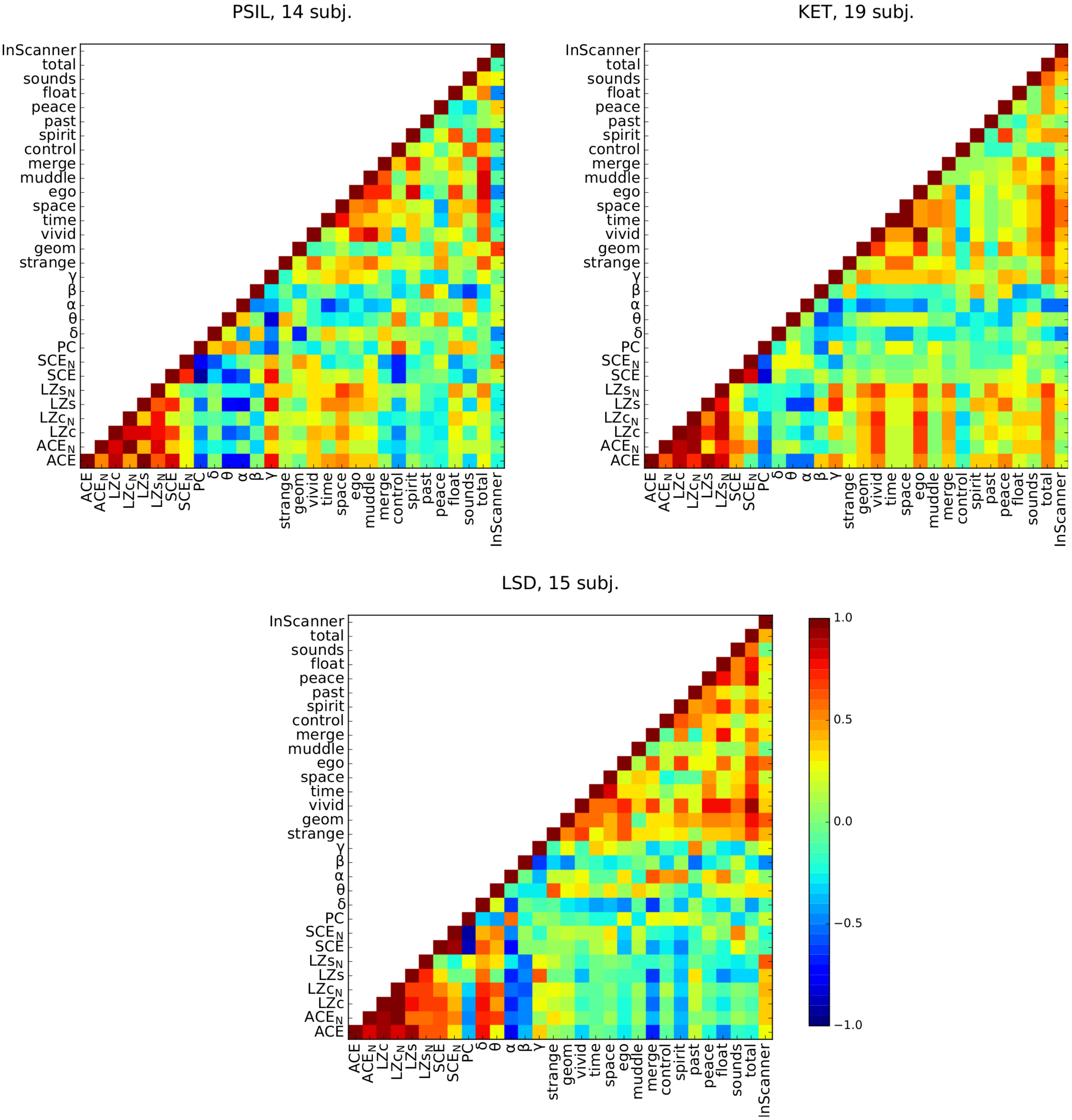

Figure S1: **Full correlations across measures and questionnaire answers.** For each drug, the Pearson correlation, r, of the score difference between drug and placebo condition (averaged across trials) of any measure (subjective rating) pair across subjects is shown. See main text for details.
